# Supercritical Carbon Dioxide and Its Potential as a Life-Sustaining Solvent in a Planetary Environment

**DOI:** 10.3390/life4030331

**Published:** 2014-08-08

**Authors:** Nediljko Budisa, Dirk Schulze-Makuch

**Affiliations:** 1 Department of Chemistry, Technical University Berlin, Müller-Breslau-Straße 10, D-10623 Berlin, Germany; 2 Center for Astronomy and Astrophysics, Technical University Berlin, Hardenbergstraße 36, D-10623 Berlin, Germany; 3 School of the Environment, Washington State University, Pullman, WA 99164, USA

**Keywords:** anhydrous solvents, biotransformations, carbon dioxide, enzymes, geochemistry, habitat, molecular memory, organic solvents, scCO_2_

## Abstract

Supercritical fluids have different properties compared to regular fluids and could play a role as life-sustaining solvents on other worlds. Even on Earth, some bacterial species have been shown to be tolerant to supercritical fluids. The special properties of supercritical fluids, which include various types of selectivities (e.g., stereo-, regio-, and chemo-selectivity) have recently been recognized in biotechnology and used to catalyze reactions that do not occur in water. One suitable example is enzymes when they are exposed to supercritical fluids such as supercritical carbon dioxide: enzymes become even more stable, because they are conformationally rigid in the dehydrated state. Furthermore, enzymes in anhydrous organic solvents exhibit a “molecular memory”,* i.e.*, the capacity to “remember” a conformational or pH state from being exposed to a previous solvent. Planetary environments with supercritical fluids, particularly supercritical carbon dioxide, exist, even on Earth (below the ocean floor), on Venus, and likely on Super-Earth type exoplanets. These planetary environments may present a possible habitat for exotic life.

## 1. Supercritical CO_2_ and Its Special Properties as a Supercritical Fluid

**Figure 1:**
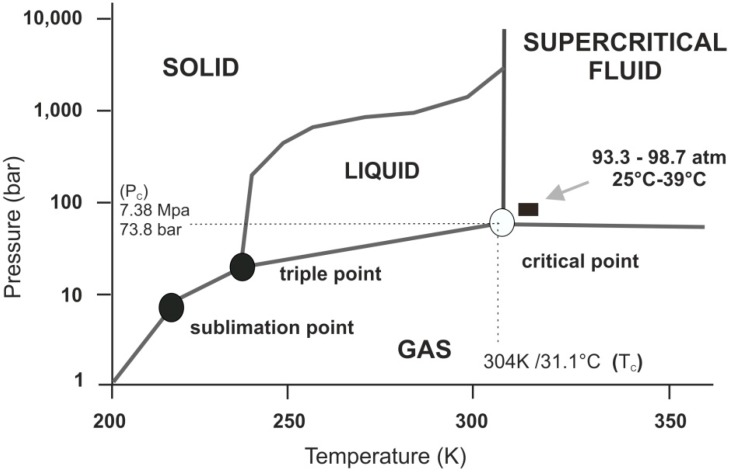
Schematic p-T phase diagram of CO_2_. Note if the temperature and pressure of a substance are both higher than Tc and Pc for a particular substance, the substance is defined as a supercritical fluid. Carbon dioxide has four distinct phases; the standard solid, liquid and gas phase as well as the supercritical phase. Carbon dioxide transitions to supercritical phase occur relatively readily at the critical points of 7.38 MPa, 304 K/31.1 °C and 73.8 bar.

Supercritical fluids (SCFs) differ quite significantly compared to the properties of real fluids. For example, supercritical water is relatively non-polar and acidic [1]. Supercritical fluids cannot be defined as a liquid or as a gas but as a substance in a state (“supercritical state”) above its critical temperature (T_C_) and critical pressure (P_C_). For example, supercritical carbon dioxide (scCO_2_; critical point: 7.38 MPa, 304 K/31.1 °C and 73.8 bar) is a nonpolar medium with large quadrupolar moment [2]. Its density can be changed as a function of temperature and pressure [3]. At critical pressure, its compressibility is maximized, and small changes to thermal parameters can lead to large changes in its local density (Figure 1). 

Not surprisingly, SCFs as non-aqueous solvents for enzyme-catalyzed reactions have gained the attention of enzymologists since the 1980s and have been employed in a variety of biotechnological applications due to their numerous advantages [4]. Enzymes are not only able to function in SCFs but they also display interesting novel properties such as altered substrate specificity and enantio-selectivity, suppression of side-reactions, increased stability, and “molecular memory” [5]. In general, SCFs differ from ordinary solvents in having both liquid-like solubilizing capacities whereas retaining high diffusivities and low viscosities of the gas phase. Near the critical point, small changes in temperature or pressure lead to significant changes in solubility, partition coefficient, dipole moment and the dielectric constant. It is relatively easy to control these properties, because small changes in pressure or temperature near the critical point can alter the reactivity in biochemical processes as the solvent strength of a supercritical fluid can be varied by changing pressure and temperature [6]. 

The change in properties from subcritical fluid to supercritical state is especially noteworthy for the common compounds water and carbon dioxide. These include: (1) high solubility of gases within supercritical mixtures, (2) miscibility of gases such as O_2_ and H_2_ in supercritical fluids, (3) high diffusion rates and variable density, and (4) high dissolving power [1,7]. As a conclusion, Ikushima advanced the case for supercritical fluids as an appropriate medium for chemical and biochemical processes under certain conditions [6].

Here, we focus on supercritical carbon dioxide (scCO_2_), which attracted particular attention in research and technology due to its “green” (*i.e.*, sustainable) properties. ScCO_2_ is chemically relatively inert (for example, it is “immune” to free radical chemistry) and is a low-toxicity aprotic solvent [8]. Unlike water, scCO_2_ is an easily accessible supercritical regime (7.38 MPa, 304 K/31.1 °C and 73.8 bar) and, as a solvent, is miscible with both fluorous and organic materials [9,10] (Figure 1). Also, carbon dioxide is at the maximum oxidation number of carbon (+IV; chemically fully oxidized state) and therefore is inert towards further oxidation (*i.e.*, non-flammable). Supercritical carbon dioxide can thus serve as solvent for “difficult” chemical transformations, such as the direct reaction of hydrogen and oxygen to form hydrogen peroxide [11] or various selective free-radical reactions [3].

## 2. Enzyme Activity in Anhydrous Organic Solvents Including scCO_2_

Organic solvents are usually volatile carbon containing compounds present in liquid form at room temperature. On the other hand, supercritical fluids such as scCO_2_ can provide environments even more remote from water than organic solvents [12], but share many common features with organic solvents. Both represent a medium in which the vast majority of industrially-relevant biotechnological synthetic enzyme reactions take place as many water-insoluble substrates can be transformed by enzymes in non-aqueous media [13]. However, organic solvents are also extremely toxic for living cells, as they are capable of disrupting the cell membrane, which results in the leakage of macromolecules including RNA and proteins [14]. Curiously, organic-solvent-tolerant bacteria such as various *Pseudomona*s strains, especially *Pseudomonas putida* can withstand such harsh environments due to the presence of various adaptive mechanisms (e.g., they are able to change the chemical composition of their membranes, in addition to other adaptation techniques) [15,16].

Enzymes of terrestrial organisms need a specific amount of bound water to be active [17]. The activity of a majority of enzymes is decreased upon transfer to non-aqueous solvents. The inactivation mechanism most probably include disruptive changes in the active site, blockage of substrate access, unfavorable substrate desolvation, and effects of transition state destabilization and restriction of conformational mobility [18]. In fact, enzymes in anhydrous environments are very rigid and through this property alone their activity is usually diminished. Water is needed for flexibility as a molecular lubricant and essential parts of the enzyme surface must be hydrated to allow catalysis to occur [19]. The three-dimensional structure of enzymes is dramatically altered under extremely dehydrating conditions, causing their denaturation and a consequent loss of their activity. However, if the conditions are less adverse, the protein structure may largely be retained. For example, scCO_2_ may dissolve from 0.3% to 0.5% (w/w) water, depending on the pressure and temperature [20].

Kalibanov was among the first to realize that the water bound to the enzyme determines the catalytic activity rather than the total water content of the system [5]. In other words, the enzyme itself is “not interested” for more than a few water molecules or a small hydrate layer to develop optimal or even maximal activity. The optimum water content required for a particular biotransformation depends on the enzyme and the solvent [21]. Therefore, to maintain biocatalysis abilities in terrestrial organisms in adverse solvent environments such as scCO_2_ or any other non-aqueous media or supercritical fluids, it is of vital importance to have a minimum water content/solubility [22]. Completely dry enzymes are inactive—a threshold value of about 0.2 g H_2_O/g enzyme is generally accepted [23]. The role of this “structural” water is not only to maintain the enzyme’s structure, but also to facilitate non-covalent bonding and disruption of hydrogen bonds during catalysis, which has a very significant influence on the reaction kinetics. On the other hand, minor structural changes may induce an alternative active protein state with altered enzyme activity, specificity and stability. For example, supercritical carbon dioxide as a solvent can accelerate mass-transfer of certain enzyme reactions [9].

**Figure 2:**
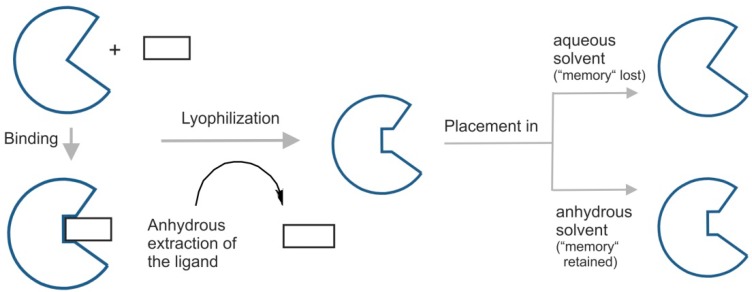
Effect of scCO_2_ as an anhydrous solvent on biochemical systems, specifically in regard to “enzyme memory”. Lyophilization of enzymes (freeze-drying or cryo-desiccation) in organic solvents can enable enzymes to “remember” the exposure to a ligand—a phenomenon known as ‘ligand imprinting” or “ligand-induced enzyme memory” [12]. The induction of enzyme memory requires considerable conformational flexibility in the protein. High conformational rigidity present in nearly all anhydrous environments is the basic requirement for appearance and retention of “memory”. For that reason, simple addition of smaller amounts of water is sufficient enough to erase the “memory” effect.

A particularly notable feature of enzymes after being dissolved in an anhydrous apolar solvent and being in contact with a competitive inhibitor is the capacity to retain the state induced by the ligand (Figure 2). This phenomenon, known as ligand imprinting, bio-imprinting or ligand-induced enzyme memory, was first reported by Russell and Klibanov [24]. They observed that the activity of subtilisin in n-octane was enhanced when it was lyophilized from a solution containing competitive inhibitors (that were subsequently removed). It has been suggested that this kind of effect may be related to a “conformation memory” similar to the “pH memory” phenomenon seen in many enzymes [17]. That is, in organic solvents including scCO_2_, the catalytic activity of enzyme reflects (*i.e.*, the enzyme “remembers”) the pH (*i.e.*, last ionization state) of last aqueous solution it was exposed to [12]. In this way, enzyme properties in a particular solvent are dependent on their history.

## 3. Supercritical Carbon Dioxide in Biochemical Transformations

**Figure 3:**
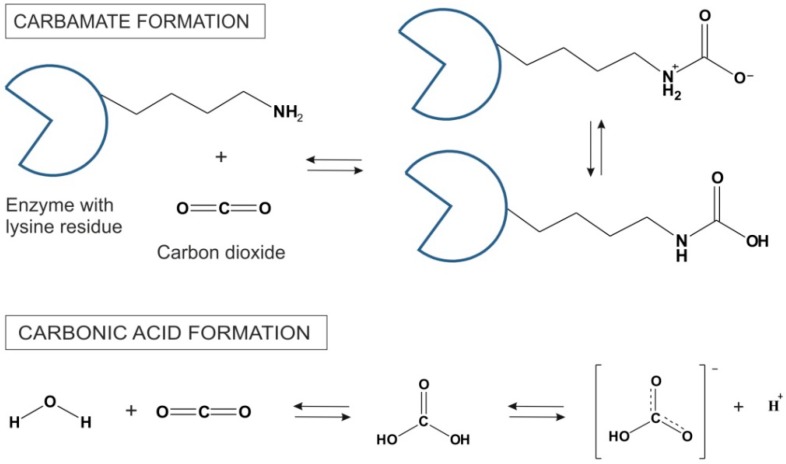
Carbamate synthesis in reaction between CO_2_ and lysine side chains on the surface of an enzyme and the formation of carbonic acid and its dissociation to the bicarbonic anion in scCO_2_ [9].

The main advantages of scCO_2_ for biochemical transformations include increased catalytic activities as a result of improved mass transfer, higher selectivities and strong suppression of side reactions [6]. However, the low dielectric constant of scCO_2_ in the liquid state (≈1.5) might require even higher pressures for certain classes of substances to be efficiently dissolved, whereas scCO_2_ as a Lewis acid is prone to react with chemical functional groups such as amines. Since enzyme surfaces are generally decorated lysine side chains, many free amine-groups are exposed to the solvent. These amine groups form carbamates with CO_2_ in a highly exothermic reaction [25]. The mechanism of CO_2_ reversible formation of carbonic acid is shown in Figure 3. The carbamates are stable at low temperatures. However, at higher temperatures CO_2_ is removed and amine function of lysine side chains is restored. Beside lysine residues, the imidazole side chain of histidine on the enzyme surface is also believed to have the capacity to participate in carbamoylation reaction [26]. In some cases, these reactions in scCO_2_ can lead to enzyme deactivation, whereas in some other instances, carbamate formation is believed to lead to changes in enzyme catalytic features (e.g., enhanced stereoselectivity). For example, Mase and co-workers speculated that a conformational change in enzyme due to carbamate formation provides good enantiomeric excess in lipase-catalyzed desymmetrization of 1,3-propanediacetate [27]. On the other hand, scCO_2_ can serve as a temporary protecting group in ruthenium-catalyzed metathesis as well as in rhodium-catalyzed hydroamino-methylations [23] and references cited therein.

Since scCO_2_ has the tendency to strip structurally relevant water (both external and internal) from the enzyme leading to deactivation, it is necessary to add a small amount of water to the substrates, which can reverse the deactivation and restore the enzyme activity. The preservation of the enzyme structure and optimal activity depend on the presence of small amounts of water in the supercritical dispersing medium. When the protein is at least partially hydrated, water molecules bind to specific sites on the enzyme surface and prevent carbon dioxide from penetrating into its catalytic core [4]. In this context, another property of scCO_2_ is that it can interfere with the catalytic activity of the enzyme by lowering the pH of the structural water (*i.e.*, microaqueous environment or hydration layer associated with the enzyme) present in scCO_2_.

Major classes of enzymes used for biocatalytic synthesis in scCO_2_ are lipases, which are used for enantioselective (trans)-esterifications and esterifications of fatty acids. Some other enzymes used in scCO_2_ are thermolysin, several phosphatases, dehydrogenases, oxidases, amylases and decarboxylases for CO_2_-fixation [4]. As scCO_2_ exhibits a liquid-like density and a gas-like viscosity (scCO_2_ liquid viscosity is only 1/10 that of water [9]) any changes in the physical properties of supercritical CO_2_ by changing the pressure may induce an alternative active protein state with altered enzyme activity, specificity and stability [23]. The catalytic features of lipases in scCO_2_ are quite well understood: water and supercritical carbon dioxide coat the protein surface in a heterogeneous manner. Thereby, surface-exposed hydrophilic residues bind water, whereas carbon dioxide solvates surface-exposed hydrophobic residues (co-solvent effect). The substrate-binding region of the lipase is exposed to carbon dioxide, which facilitates diffusion of large apolar substrates (*i.e.*, fatty acids) into active sites (hydrophobic tunnel), while preserving the functional structure of the enzyme [19]. Leitner pointed out that enzymes in scCO_2_ would not be considered analogous to an enzyme in solution, but rather behave as heterogeneous catalysts where diffusion of reactants in and out of the enzyme is not rate limiting [28].

The majority of the reactions studied in scCO_2_ are hydrolytic reactions, which require the presence of catalytic traces of water (“catalytic water”). This certainly does not disqualify scCO_2_ as a potential solvent for life. For example, for nearly all oxidation-reduction reactions catalyzed by enzymes in water “catalytic amounts” of metals, either tightly bound to the enzyme or taken up from bulk solvent along with the substrate, are required. In fact, almost one-third of all known enzymes require one or more metal ions for catalytic activity, usually in order to catalyze certain reactions by reversible changes in the bound metal ion’s redox state. The absolute necessity of traces of inorganic ions for enzymatic activity for so many enzymes in Earth’s biochemistry certainly does not disqualify water as life solvent.

## 4. Supercritical Carbon Dioxide, a Biological Agent?

ScCO_2_ has a relatively low critical pressure and temperature [4] and is traditionally used as an alternative food-safety sterilization procedure/treatment, because it is a non-thermal process capable of inactivating microorganisms at relatively moderate pressures such as 7.3–50.0 MPa at relatively low temperatures [29]. There is still no generally accepted view about the mechanism of the bactericidal activity of scCO_2_ [23], which may be related to pressure, temperature, and exposure time. For example, higher pressure enhances CO_2_ solubility and facilitates the acidification of the cellular interior [30]. On the other hand, scCO_2_ is believed to increase the fluidity of the cell membrane, enhancing its permeability and facilitate extraction of membrane components such as phospholipids [31]. This should not be surprising as the surface tension in carbon dioxide is much lower than that of water, whereas the diffusivity of solutes in scCO_2_ is markedly higher (low viscosity!). Thus, scCO_2_ can much easier penetrate complex molecular geometries and assemblies (e.g., cell membranes) than subcritical fluids can. Georgiou and Deamer have suggested supercritical fluid-mediated lipid extraction technology as the main method of choice in the search for biomarkers for extraterrestrial life. In particular, lipid-derived hydrocarbons as biomarkers must have a specific compositional pattern (e.g., odd/even number of carbon atoms in their chains, branching and unsaturation levels) that originates exclusively from biogenic processes [32].

On the other hand, scCO_2_ may be an appropriate medium for biochemical processes under certain conditions. Ikushima [6] reported the synthesis of esters from acryl donors and terpene alcohols by lipase in *Candida cylindracea*, which in supercritical carbon dioxide caused drastic conformational changes that enabled active sites to catalyze stereoselective synthesis. The reactivity was found to be susceptible to small changes in pressure or temperature near the critical point of the supercritical fluid. Shkrob and Sauer [33] showed that high mobility CO_2_-multimer anions in supercritical carbon dioxide form stable complexes with water, aliphatic alcohols, alkyl halides, and alkyl nitriles. Matsuda and co-workers have demonstrated that the alcohol dehydrogenase from *Geotrichum candidum*, active in scCO_2_ at 35 °C and 10 MPa is capable of catalyzing the asymmetric reduction of various ketones [34]. The survival of the cells and preservation of enzyme activity under these conditions were explained by the water pool of the cytoplasm of cells in the resting state [35]. Through the solvent is in this case no longer scCO_2_, it shows that the application of scCO_2_ is not incompatible with life processes. Various enzymatic reactions were studied using bacterial species such as *Bacillus megaterium*, *Geotrichum candidum* or *Aureobasidium pullulans* (for detailed review see [23]).

An example of a planetary environment where supercritical CO_2_ might support living processes was reported by Schulze-Makuch and Irwin [7]. They pointed to the discovery of subsurface accumulations of liquid carbon dioxide under Earth’s oceans [36]. The low density liquid CO_2_ has been found to be trapped by a surface rock layer and sub layer cap of CO_2_ hydrate (CO_2_ x 6H_2_O). As the density of liquid CO_2_ increases with depth, it becomes denser than sea water, which opens up the possibility of many reservoirs of supercritical carbon dioxide on the sea floor [37]. Most intriguing was the detection of 10^7^ cells/ml at the fluid CO_2_/CO_2_-hydrate interface [36], which is quite remarkable given the potentially hostile nature of CO_2_ [38]. Since CO_2_ is a very common compound in planetary atmospheres, including our neighboring planets Venus and Mars, scCO_2_ is expected to occur in a variety of planetary environments. 

One location where CO_2_ should occur in the supercritical state is in the near-subsurface of Venus. Venus is nearly as massive as Earth and has an atmosphere of 92 bar, which mostly consists of CO_2_. Thus, it would be a great natural laboratory for testing the properties of scCO_2_. An interesting twist is that Venus was located in the habitable zone of our Solar System in its early history. Schulze-Makuch and co-workers [39,40] suggested the presence of an early biosphere on the surface of this planet, before a run-away greenhouse effect made all life near the Venusian surface all but impossible. However, perhaps some biomarkers from this earlier biosphere could have been preserved in the supercritical CO_2_. [32] The importance of scCO_2_ extends far beyond Venus though, since many of the detected exoplanets are Super-Earths, with 10 or more earth masses. All these high pressure environments are locations where we might expect scCO_2_, given its common occurrence. Certainly, scCO_2_ deserves more attention not only for its biochemical and biotechnological applicability, but also as potential life-sustaining solvent in exotic planetary environments. 

## 5. Conclusions

When we consider scCO_2_ as a potential life-sustaining medium, we need to free us from the classical anthropocentric approach that defines water as the exclusive and sole solvent of life. Doubtlessly, putative life on a planet with scCO_2_ alone or in a mixture with water (to various extents) would be based on a different biochemistry, including different organization of boundary conditions (membranes), metabolic pathways, informational and energy flows as well as different polymers with informational and catalytic functions. The problem of increased conformational rigidity of enzymes in scCO_2_, known from Earth’s biochemistry, should not be taken dogmatically, particularly the notion that hyperthermophile proteins, due to enhanced conformational inflexibility in the folded native state, cannot achieve high enzyme activity (at lower temperatures) with a rigid active site(s) [41]. Proteins elsewhere might have a different composition other than the canonical 20 natural amino acids and the monomeric building blocks of enzymes might have a different backbone and side chain chemistries. Indeed, it is well documented that hyperthermophilic proteins are capable to perform extremely high catalytic activity (even at low temperature) that can salvage short-lived substrates or metabolically unstable intermediates in a rigid active site in the context of hyperstable protein scaffolds [41].

Therefore, carbon dioxide is not just a “greenhouse gas” but also an abundant molecule with many potential applications for life. In the supercritical phase, it is an aprotic solvent miscible with a variety of organic liquids and some bacteria and their enzymes are active in this solvent. Planetary environments with supercritical carbon dioxide exist under the seabed of the Earth, on Venus, and probably on exoplanets such as Super-Earths.
